# The utilization of appropriate osteoporosis medications improves following a multifaceted educational intervention: the Canadian quality circle project (CQC)

**DOI:** 10.1186/1472-6920-9-54

**Published:** 2009-08-06

**Authors:** George Ioannidis, Alexandra Papaioannou, Lehana Thabane, Amiram Gafni, Anthony Hodsman, Brent Kvern, Aleksandra Walsh, Famida Jiwa, Jonathan D Adachi

**Affiliations:** 1 Department of Medicine, McMaster University, Hamilton, Canada; 2 Department of Clinical Epidemiology and Biostatistics, McMaster University, Hamilton, Canada; 3 Director of Biostatistics, Father Sean O'Sullivan Research Centre and Centre for Evaluation of Medicines, St Joseph's Healthcare, Hamilton, Canada; 4 Department of Medicine, University of Western Ontario, London, Canada; 5 Department of Family Medicine, University of Manitoba, Winnipeg, Canada; 6 Procter & Gamble Pharmaceuticals, Toronto, Canada; 7 Osteoporosis Canada, Toronto, Canada

## Abstract

**Background:**

Osteoporosis is a serious but treatable condition. However, appropriate therapy utilization of the disease remains suboptimal. Thus, the objective of the study was to change physicians' therapy administration behavior in accordance with the Osteoporosis Canada 2002 guidelines.

**Methods:**

The Project was a two year cohort study that consisted of five Quality Circle (QC) phases that included: 1) Training & Baseline Data Collection, 2) First Educational Intervention & First Follow-Up Data Collection 3) First Strategy Implementation Session, 4) Final Educational Intervention & Final Follow-up Data Collection, and 5) Final Strategy Implementation Session. A total of 340 family physicians formed 34 QCs and participated in the study. Physicians evaluated a total of 8376, 7354 and 3673 randomly selected patient charts at baseline, follow-up #1 and the final follow-up, respectively. Patients were divided into three groups; the high-risk, low-risk, and low-risk without fracture groups. The generalized estimating equations technique was utilized to model the change over time of whether physicians

**Results:**

The odds of appropriate therapy was 1.29 (95% CI: 1.13, 1.46), and 1.41 (95% CI: 1.20, 1.66) in the high risk group, 1.15 (95% CI: 0.97, 1.36), and 1.16 (95% CI: 0.93, 1.44) in the low risk group, and 1.20 (95% CI: 1.01, 1.43), and 1.23 (95% CI: 0.97, 1.55) in the low risk group without fractures at follow-up #1 and the final follow-up, respectively.

**Conclusion:**

QCs methodology was successful in increasing physicians' appropriate use of osteoporosis medications in accordance with Osteoporosis Canada guidelines.

## Background

Osteoporosis, a chronic debilitating disease, is a major clinical concern in postmenopausal women. Nearly any bone can fracture as a result of increased bone fragility. The most frequent sites of osteoporotic fracture take place at the hip, spine and wrist [1-4]. The major complications of fracture include increased mortality, reduced health related quality of life and higher care expenditures [5-14].

While osteoporosis may be a potentially crippling condition, it can be identified early during the course of the disease and effective therapy can be administered. After a patient has been diagnosed with osteoporosis, treatment should be initiated, given that there are many proven and effective modern pharmacological therapies for the prevention and treatment of the disease [15]. However, despite the consequences of osteoporosis, the management of the disease is less than optimal. For example, Papaioannou et al. [16] evaluated patients re-fracture rates following hip fractures in 527 patients aged 50 years. The frequency of preventative treatments for osteoporosis was low given the risk of re-fracture. None of the patients were given bisphosphonates or hormone therapy and only 18% were given calcium and vitamin D. Furthermore, in a study that obtained data on osteoporosis drug therapy in 112 patients who were diagnosed as having minimal trauma forearm fractures, results indicated that a total of 42 patients (38%) were receiving either hormone replacement therapy or using a bisphosphonate. Moreover, a search conducted from a claims database in 30 states by Freedman et al. identified 1162 women, age 55 years or older who had a distal radial fracture. Of these patients, only 22.9% were treated with at least one of the medications approved for treatment of established osteoporosis [17]. Finally, data from the National Ambulatory Medical Care Survey from 1993 to 1997 were examined for evidence of treatment for osteoporosis or vertebral fracture during visits by women 60 years of age and older to family physicians. Results indicated that appropriate drug therapies including calcium and vitamin D were offered to only 36% of the diagnosed patients [18].

Given the management care gap and the consequences of osteoporosis, it is important the physicians treat patients with osteoporosis appropriately. To reduce this care gap, the Quality Circle (QC) project, a multifaceted integrated disease management process strategy was developed and implemented (19, 20). The objective of the study was to determine and reduce the care gap in treating patients with osteoporosis. We hypothesize that family physicians' use of appropriate therapy will change in accordance with the Osteoporosis Canada guidelines as a result of their enrollment in quality circles.

## Methods

The Canadian QC Study has been described elsewhere [19,20]. Briefly, the project was designed to collect, analyze, and distribute information on physician practices in the diagnosis and treatment of osteoporosis so that the deficiencies in disease management may be recognized and beneficial actions may be developed and put into practice through QCs, a multifaceted osteoporosis educational intervention strategy involving small group meetings.

Family physicians were recruited from seven provinces across Canada. Three groups of physicians were selected and participated in the study. Facilitators were local family physicians recruited to lead the study meetings and were chosen because of their involvement in continuing professional development. Circle members were family physicians selected from specific geographical regions across Canada to form the QCs. A maximum of 15 physicians were enrolled in each QC. An osteoporosis specialist was recruited for each circle. The assignment of the osteoporosis specialist was to guide circle members on complicated clinical matters.

All participating physicians provided written informed consent. Physicians were selected because of their interests in osteoporosis. The study was approved by Health Research Ethics Boards across Canada. The study was sponsored by research grants from the Ontario College of Family Physicians and the Alliance for Better Bone Health (Procter & Gamble Pharmaceuticals Canada Inc. and sanofi-aventis Pharma Inc.).

### Overview of Project Phases

The project consisted of five QC phases that included: 1) Training & Baseline Data Collection, 2) First Educational Intervention & First Follow-Up Data Collection 3) First Strategy Implementation Session, 4) Final Educational Intervention & Final Follow-up Data Collection, and 5) Final Strategy Implementation Session [19]. The duration of the study consisted of 2 one year segments.

Our educational intervention consisted of eight key components and consisted of 1) audit and feedback, 2) interactive small group discussions 3) use of opinion leaders, 4) remainders, 5) multiprofessional collaboration with osteoporosis specialists, 6) nominal financial reimbursement to circle members, 7) patient medicated interventions 8) and educational material [19,20].

The QC educational intervention workshop was created by a committee consisting of members from Osteoporosis Canada, Ontario College of Family physicians, leading physicians and scientists, and industry partners. The 2002 Osteoporosis Canada guidelines were used as the main evidence-based reference for the program. The workshop was administered twice and took 90–120 minutes to complete [19,20].

### Osteoporosis Canada Guideline Recommendations

Appropriate therapy was defined based on the Osteoporosis Canada 2002 guidelines [15]. According to the guidelines, patients should be given osteoporosis therapy if they have at least one of the following criteria: osteopenia (t-score < -1.5) and prior fragility fracture, osteopenia and at least one other major (excluding prior fracture) or two minor risk factors for future fracture, or osteoporosis defined as a bone mineral density t-score of less than -2.5 regardless of risk factor status. Treatment should not be administered if the patient has osteopenia and only one minor or no risk factors for fracture, or has normal bone mineral density or has not had a bone density test regardless of their risk factor status [15].

### Patients & Therapy

Eligible patients were enrolled based on the following criteria: women 55 years of age and older, were known to the clinician, and had at least two appointments in the 24 months before enrollment. The screening methods for choosing eligible patients were carried out by the clinic nurse to circumvent the potential of physician bias. Patient screening was designated for one day each week and was repeated for 8 weeks. At the end of each recruitment day, the clinic nurse used the day's visit schedule to randomly select the medical charts of three or four patients that met the eligibility criteria of the study. A total of 25 different patient charts were chosen for evaluation.

After making the selection, the clinic nurse inserted the patient questionnaire into each patient chart and the physician completed the form to ascertain how they treated osteoporosis. Based on the questionnaire responses, physician profiles were generated that showed how individual physicians treated patients in their own practices. The profiles permitted anonymous comparisons of individual circle member data with their peers in their circle and with all the participating physicians in the project. The physicians' profiles were than compared to the Osteoporosis Canada guidelines.

For the current analysis, patients were divided into three groups. The high risk group was defined as patients with osteoporosis based on a bone mineral density t-score of less than -2.5 regardless of risk factor status, or osteopenia and prior fragility fracture, osteopenia and at least one other major risk factor (excluding prior fracture), or two minor risk factors for future fracture (high risk group definition base on Osteoporosis Canada guidelines). The low risk group was defined as patients with osteopenia and no prior fracture, osteopenia and at most one minor risk factor for fracture, or normal or no bone mineral density measurements regardless of risk factor status (low risk group definition base on Osteoporosis Canada guidelines). Thus, individuals at low risk may have experienced a fracture and still be considered low risk if the patient had normal bone density or did not have a bone density scan. The low risk no fracture group was defined as patients with osteopenia and no prior fracture, osteopenia and at most one minor risk factor for fracture, or normal or no bone mineral density measurements regardless of risk factor status (excluding patients with fracture).

The treatments that were evaluated in the current study included alendronate, calcitonin, etidronate, hormone replacement therapy, parathyroid hormone (PTH), raloxifene, or risedronate. To avoid the use of hormone replacement therapy for other conductions we excluded all patients on this therapy if they had a history of significant vasomotor symptoms.

### Statistical Analysis

For our primary analysis, the generalized estimating equations [21] technique utilizing an exchangeable correlation structure was used to model the change in the appropriate use of osteoporosis therapies over time (baseline, 1^st ^follow-up, and final follow-up). The generalized estimating equations method was performed to factor in the clustered or correlated nature of the data given that individuals treated by the same family physician will be managed similarly (clustered variable is the physician). For each model, the patient is the unit of analysis and the family physician is the unit of inference. Odds ratios (OR) and corresponding 95% confidence intervals (CI) are calculated. Goodness-of-fit of each model was determined by the technique created by Horton et al. [22].

The change in the appropriate use of osteoporosis therapies over time was evaluated in the high risk group, the low risk group and the low risk group without prior fractures. For the high risk group, the administration of any of the above listed therapies was defined as appropriate. If treatment was not provided, it was considered inappropriate. For the low risk group, no treatment was deemed appropriate, whereas the administration of therapy was inappropriate. Thus, the outcome was a binary variable (1 = appropriate use, 0 = inappropriate) and the primary independent variable was time (baseline, follow-up #1, and final follow-up). In addition, results were adjusted for the patient's age (≤65/> 65 years), prior fracture status at the hip, spine or wrist (yes/no), family history of fragility fracture (yes/no), early menopause (yes/no), other major risk factors for fracture (yes/no), and two or more minor risk factors for fracture (yes/no).

To assess the robustness of the results produced by the generalized estimating equations analyses (our primary analysis), a random coefficient (mixed effects model) and standard logistic regression analyses were conducted [23]. All statistical analyses were performed using the SAS/STAT (version 9.1; SAS Institute Inc., Cary, North Carolina, USA) software package running on Windows XP Professional. The criterion for statistical significance was set at α = 0.05.

## Results

A total of 340, 301 and 162 family physicians from across Canada formed 34, 31, 27 QCs at baseline, follow-up 1 and the final follow-up, respectively. Family physicians evaluated a total of 8376, 7354 and 3673 randomly selected patient charts at baseline, follow-up #1 and the final follow-up, respectively. Table 1 displays patient characteristics. During the three data collections phases over 60% of patients were 65 years of age and older. In addition, 10.5 (883/8376), 12.6 (923/7354), and 11.8% (434/3673) of patients had prior fractures; 63.2 (5292/8371), 66.2 (4849/7328), and 75.3% (2755/3657) of patients had a BMD test; and 30.5 (2555/8376), 32.9 (2417/7354), and 37.9% (1393/3673) of patients received bisphosphonate therapy during baseline, follow-up #1 and the final follow-up, respectively.

**Table 1:** Patient characteristics at baseline, Follow-up #1 and Final Follow-up.

Patient characteristics	Baseline % (n)	Follow-up #1 % (n)	Final Follow-up % (n)
>65 years	64.2 (5337/8317)	60.5 (4440/7341)	62.3 (2286/3670)
Prior hip fracture	1.8 (152/8366)	2.6 (187/7344)	2.4 (87/3667)
Prior wrist fracture	4.2 (353/8367)	5.3 (392/7345)	4.9 (180/3665)
Prior spine fracture	5.8 (486 (8365)	6.6 (481/7339)	7.1 (259/3663)
High risk patients	76.0 (6367/8376)	75.7 (5569/7354)	78.2 (2871/3673)
			
BMD tests			
None	36.8 (3079/8317)	33.8 (2479/7328)	24.7 (902/3657)
Normal	17.8 (1490/8317)	17.7 (1295/7328)	19.3 (704/3657)
Osteopenia	24.2 (2028/8317)	31.1 (1272/7328)	35.9 (1313/3657)
Osteoporosis	21.2 (1774/8317)	17.4 (1272/7328)	20.2 (738/3657)
			
Any therapy	38.3 (3210/8376)	39.4 (2984/7354)	43.5 (1596/3673)
Risedronate	10.8 (908/8376)	13.7 (1008/7354)	19.9 (732/3673)
Alendronate	12.5 (1050/8376)	13.3 (979/7354)	13.0 (479/3673)
Etidronate	7.1 (597/8376)	5.9 (430/7354)	5.0 (182/3673)
Hormone replacement	6.3 (509/8025)	5.5 (389/7106)	4.3 (155/3605)
Raloxifene	2.1 (172/8376)	1.7 (123/7354)	1.6 (60/3673)
PTH	0.1 (6/8376)	0.0 (3/7354)	0.2 (7/3673)
Calcitonin	1.0 (83/8376)	0.6 (45/7354)	0.6 (22/3673)

### Appropriate Therapy Administration: Generalized Estimating Equations

Results revealed that family physicians use of appropriate therapy increased following the educational interventions in accordance with the Osteoporosis Canada 2002 guidelines. Compared with baseline, more high risk patients were administered appropriate therapy. At the final follow-up visit, approximately 41% of patients were given appropriate therapy. In addition, compared with baseline, fewer low risk patients were administered therapy. At the final follow-up visit, approximately 86% of patients at low risk without fractures were given appropriate therapy (Table 2).

**Table 2:** The use of appropriate therapy in the High risk, Low risk and Low risk without fractures group at baseline, Follow-up #1 and Follow-up #2*

	Baseline % (n)	Follow-up #1 % (n)	Final follow-up % (n)
Appropriate therapy			
High risk group	34.7 (1040/2996)	40.1 (1243/3098)	41.2 (735/1784)
Low risk group	81.9 (2874/3506)	83.4 (2052/2460)	84.3 (782/928)
Low risk group without fractures	83.4 (2778/3332)	86.1 (1956/2272)	86.5 (763/882)

* The high risk group was defined as patients with osteopenia and prior fragility fracture, osteopenia and at least one other major (excluding prior fracture) or two minor risk factors for future fracture, or osteoporosis defined as a bone mineral density t-score of less than -2.5 regardless of risk factor status. The low risk group was defined as patients with osteopenia and no prior fracture, osteopenia and at most one minor risk factor for fracture, have normal or no bone mineral density measurements regardless of risk factor status (guidelines). The low risk no fracture group was defined as patients with osteopenia and no prior fracture, osteopenia and at most one minor risk factor for fracture, have normal or no bone mineral density measurements without a prior fracture. Appropriate therapy was defined as any treatment (including alendronate, calcitonin, etidronate, hormone replacement therapy, PTH, raloxifene, or risedronate) administered to high risk patients and no treatment administered to low risk patients.

The odds of appropriate therapy significantly increased following the educational interventions in the high risk group and the low risk group without fractures by 20 to 41% (Table 3). No significant change was found in the appropriate use of therapy in the low risk group following the study (Table 3). The goodness-of-fit test for the analyses varied from 0.0002 to 0.319.

**Table 3:** Appropriate therapy during the study determined by the Generalized estimating equations, Random coefficient, and Logistic regression analyses.*

	**Generalized estimating equations¶**	**Random coefficient analysis¶**	**Logistic regression analysis¶**
	**Odds ratios** **(95% CI)**	**Odds ratios** **(95% CI)**	**Odds ratios** **(95% CI)**
**High risk**			
Baseline	1	1	1
Follow-up #1	**1.29** **(1.13, 1.46)**	**1.33** **(1.17, 1.50)**	**1.35** **(1.20, 1.51)**
Final follow-up	**1.41** **(1.20, 1.66)**	**1.48** **(1.27, 1.72)**	**1.44** **(1.27, 1.64)**
**Low risk**			
Baseline	1	1	1
Follow-up #1	1.15 (0.97, 1.36)	1.17 (0.99, 1.49)	**1.18** **(1.01, 1.37)**
Final follow-up	1.16 (0.93, 1.44)	1.18 (0.93, 1.46)	1.16 (0.94, 1.42)
**Low risk without fracture**			
Baseline	1	1	1
Follow-up #1	**1.20** **(1.01, 1.43)**	**1.23** **(1.03, 1.46)**	**1.22** **(1.05, 1.44)**
Final follow-up	1.23 (0.97, 1.55)	1.24 (0.96, 1.59)	**1.26** **(1.01, 1.58)**

* The high risk group was defined as patients with osteopenia and prior fragility fracture, osteopenia and at least one other major (excluding prior fracture) or two minor risk factors for future fracture, or osteoporosis defined as a bone mineral density t-score of less than -2.5 regardless of risk factor status. The low risk group was defined as patients with osteopenia and no prior fracture, osteopenia and at most one minor risk factor for fracture, have normal or no bone mineral density measurements regardless of risk factor status (guidelines). The low risk no fracture group was defined as patients with osteopenia and no prior fracture, osteopenia and at most one minor risk factor for fracture, have normal or no bone mineral density measurements without a prior fracture. Appropriate therapy was defined as any treatment (including alendronate, calcitonin, etidronate, hormone replacement therapy, PTH, raloxifene, or risedronate) administered to high risk patients and no treatment administered to low risk patients.

¶ results were adjusted for the patient's age (≤65/> 65 years), prior fracture status at the hip, spine or wrist (yes/no), family history of fragility fracture (yes/no), early menopause (yes/no), other major risk factors for fracture (yes/no), and two or more minor risk factors for fracture (yes/no). For the low risk without fracture group, the prior fracture status variable was excluded from the adjusted results.

### Appropriate Therapy Administration: Random Coefficient and Logistic Regression Analyses

Results from the random coefficient reveal similar findings to the generalized estimating equations analysis for all three groups (Table 3). However, the logistic regression analysis had a greater number of significant results. In contrast to both the generalized estimating equations and random coefficient analysis, where no significant results were found, findings from the logistic regression analysis indicated that appropriate therapy was significantly given more often during follow-up # 1 for low risk patients and during the final follow-up for low risk patients without fracture.

## Discussion

To guide physicians in treating the disease, Osteoporosis Canada has developed and circulated the 2002 Clinical Practice Guidelines for the Management of the disease [15]. Nonetheless, even with guidelines, it has been demonstrated that a care gap exist in managing patients in everyday clinical settings [16-18,24,25].

There are several potential reasons as to why osteoporosis is under-treated. For instance, family physicians may have a tendency to overlook the impact of osteoporosis given that individuals with osteoporosis are no symptoms (other than fracture), and may instead focus on other conditions they consider more severe. Physicians may lack confidence regarding the management of osteoporosis and thus are not able to provided appropriate treatment strategies, they may not be aware or may not understand the Osteoporosis Canada's management guidelines for osteoporosis that outline treatment options, or they may be discouraged by low patient adherence to their medications possibly due to poor patient education and as a consequence prescribe less often.

Given this care gap, and the availability of effective osteoporosis therapies, our study was designed to improve patient care. Following the educational interventions that included the distribution and discussion of educational materials related to the 2002 OC guidelines, the evaluation of physician profiles, an educational workshop, and facilitators led small group discussions that identify barriers to the management of osteoporosis and strategies to improve patient care, family physicians demonstrated greater odds of administering osteoporosis therapy appropriately over a two year period. The appropriate use of therapy was observed in both high and low risk patients, which revealed that physicians did not just prescribe more therapy in general, but reduced their osteoporosis prescriptions in low risk patients.

There were differences in the use of appropriate therapy between the two low risk groups (appropriate therapy increase significantly in the low risk group with no fractures and remained unchanged in the low risk group). This finding is interesting and may indicate that physicians are more willing to treat patients with fractures regardless of their bone density measurements (i.e. normal or no bone density measurement) in spite of the guidelines recommendations.

To compare the results produced by the generalized estimating equations analyses (our primary analysis), a random coefficient (mixed effects model) and standard logistic regression analyses were conducted. For the generalized estimating equations analysis, a correction is made for the within-physician correlations between the repeated measurements by assuming a working correlation structure. For the current study, the correlation between individual physicians and their patients were 0.11, 0.10, and 0.09 for the high risk, the low risk, and the low risk group without fractures, respectively. The generalized estimating equations method is robust to an assigned correlation structure; such that if a correlation structure is misspecify the analysis will still obtain consistent parameter estimates [21]. The random coefficient analysis, deals with within-physician correlations by allowing different coefficients to be random. The logistic regression analysis does not take into account the correlation and treats the data as independent [21,23].

Both the logistic and random coefficient analysis confirmed our finding with the generalized estimating equations analysis. However, conclusions drawn from the results of these methods should be viewed in the context of the models. The main concern with ordinary logistic regression is that the analysis does not take into account the within subject correlation of the physicians resulting from repeated measures. As a consequence, while the parameter estimates from the logistic regression analyses are similar to the other models, the standard errors of the estimates are generally smaller resulting in smaller confidence intervals and p-values. As a consequence, logistic analysis revealed more significant results for both the low risk and low risk without fracture groups as compared with the generalized estimating equations and random coefficient analyses.

Furthermore, it is important to realize that the regression coefficients calculated with generalized estimating equations method are population averaged. The regression coefficients calculated with random coefficient analysis can be seen as subject specific [21,23]. As a result, these two model types have different targets of inference. Given that we performed a trial concerned with changes in the mean responses over time in the study population and we were not interested in individual physician change over time, the generalized estimating equations technique is a more appropriate analysis as compared with the random coefficient method.

There are other methods available for analyzing clustered data including unweighted linear regression analysis or robust standard errors [26]. In general, analyses may be performed using individual-level data or aggregated at the cluster level. Analyses at the aggregated level are conducted using data derived from summary statistics for each cluster.

Given the large number of methods available and the fact that results may differ among techniques, it is important that investigators specify the primary data analysis in advance. However, while simulation studies show that generalizing estimating equations have the greatest statistical power among several commonly used methods for analyzing binary clustered data, there are no guidelines that evaluated what method is the most appropriate; therefore, individual investigators will have to make judgments as to which analysis to conduct [26,27].

This national study has several important strengths. For example, the project selected a large number of family physicians from across the country who examined over 19000 patients' charts from their own practices, which will improve the generalizability of our findings. Furthermore, the patient chart audits were chosen at random and did not depend on physicians self-reports, which may mirror physicians' attitudes about their practice but not reflect the true practice. Because single-component interventions have not consistently been found to transform behavior [28-30], the QC project combined various methodologies into one multifaceted intervention, which may be more useful [31,32]. In addition, physicians' behavior change observed in our study was long lasting, given that the project was conducted over a two year period.

Nonetheless, our study has some limitations. All patients examined in the project were postmenopausal women and as a consequence, the relationship between appropriate management over time may be different in premenopausal women or male patients. Furthermore, given that physician recruitment was based on the clinician's interest in women's health and osteoporosis, these members may have more knowledge in treating the disease prior to enrollment. Also, because only physicians who were interested and willing to commit to the second year of the project completed the final follow-up data collection, a lower sample of physicians reduce the power of the study to detect change over that time period. Moreover, a randomized control trial of physicians is necessary to determine the precise mechanism that the educational intervention had on physicians' behavior. Finally, it is important to consider that Osteoporosis Canada practice guidelines were developed and distributed to provide clinicians with a summary of the best evidence from clinical trials to help physicians make health care decisions regarding osteoporosis; however, clinical judgment and the patient's preference, will determine if therapy is initiated. As such, 100% compliance to the guidelines is not reasonable.

## Conclusion

In conclusion, it is vital that physicians do not prescribe medications to low risk patients and treat individuals who are at high risk for developing fractures given that several treatments have been shown to prevent fractures. The QC technique is an effective knowledge translation approach that increases family physicians' utilization of appropriate therapy in accordance with the Osteoporosis Canada 2002 guidelines. As a result of appropriate care, patients' health outcomes should improve by reducing the risk of future fractures.

## Competing interests

AP: Honoraria, grants received, or consultancies – Eli Lilly and Company, Merck Frosst, Amgen Inc, The Alliance for Better Bone Health (Procter & Gamble Pharmaceuticals and sanofi-aventis), Novartis Pharmaceuticals Corporation. AH: honoraria or consultancies – Eli Lilly and Company, Merck Frosst, NPS-Allelix, Zelos Therapeutics, Servier, Pfizer Pharmaceuticlas USA, Novartis Pharmaceuticals Corporation, The Alliance for Better Bone Health (Procter & Gamble Pharmaceuticals and sanofi-aventis), GlaxoSmithKline Consumer Healthcare. BK: honoraria or consultancies – The Alliance for Better Bone Health (Procter & Gamble Pharmaceuticals and sanofi-aventis). AW: Employee-Procter & Gamble Pharmaceuticals. JDA: Honoraria, grants received, or consultancies: – Eli Lilly and Company, Merck Frosst, Amgen Inc, The Alliance for Better Bone Health (Procter & Gamble Pharmaceuticals and sanofi-aventis), Novartis Pharmaceuticals Corporation, GlaxoSmithKline Consumer Healthcare, Servier, Roche, Servier, Wyeth.

## Authors' contributions

GI conceived the study, participated in the study design, the acquisition of data, performed the statistical analysis, and drafted the manuscript. AP made substantial contributions to the concept and design of the study, interpretation of data and critically revised the manuscript for important intellectual content. LT made substantial contributions to the statistical analysis, interpretation of data and critically revised the manuscript. AG assisted in the interpretation of the data and helped draft the manuscript. AH participated in the design of the study, the interpretation of the data and revising the manuscript. BK participated in the design of the study, the interpretation of the data and revising the manuscript. AW made substantial contributions to the concept and design of the study, and revised the manuscript. FJ made contributions to the interpretation of data and helped revised the manuscript. JDA helped conceived the study, participated in the study design, the acquisition of data, and revised the manuscript. All authors read and approved the final manuscript.

## Pre-publication history

The pre-publication history for this paper can be accessed here:


